# Fur Protein Regulates the Motility of Avian Pathogenic *Escherichia coli* AE17 Through Promoter Regions of the Flagella Key Genes *flhD*

**DOI:** 10.3389/fvets.2022.854916

**Published:** 2022-04-18

**Authors:** Lulu Niu, Weizhen Cai, Xi Cheng, Zhe Li, Jianming Ruan, Fangguo Li, Kezong Qi, Jian Tu

**Affiliations:** Anhui Province Key Laboratory of Veterinary Pathobiology and Disease Control, College of Animal Science and Technology, Anhui Agricultural University, Hefei, China

**Keywords:** Avian pathogenic *Escherichia coli*, Fur, *RyhB*, mobility, FlhD, EMSA

## Abstract

Avian pathogenic *Escherichia coli* (APEC) is an important pathogen causing several diseases in birds. It is responsible for local and systemic infections in poultry, seriously impeding the development of the poultry industry, and poses a potential risk to public health. The iron absorption regulatory protein Fur and the noncoding RNA, RyhB, that it negatively regulates are important factors in bacterial iron uptake, but the regulation of bacterial virulence genes varies greatly among different bacteria. We found that Fur is very important for the mobility of APEC. The expression of *fur* and RyhB is extensively regulated in APEC, and RyhB expression is also negatively regulated by Fur. A transcriptomic analysis showed that the genes significantly differentially regulated by Fur are related to cell movement, including pilus- or flagellum-dependent cell motility. To verify these results, we examined the effects of *fur* knockdown on cell movement by measuring the diameter of the bacteria colonies. Consistent with the RNA sequencing results, the mobility of AE17Δ*fur* was significantly reduced compared with that of the wild type, and it had almost lost its ability to move. Using an electrophoretic mobility assay, we confirmed that the Fur protein directly binds to the promoter region of the key flagellum-related gene *flhD*, thereby affecting the assembly and synthesis of the APEC flagellum. This study extends our understanding of gene regulation in APEC.

## Introduction

Avian pathogenic *Escherichia coli* (APEC) is an important pathogen causing several diseases in birds, and is mainly responsible for pathological symptoms such as pericarditis and perihepatitis (1). It is widely distributed throughout the world and often causes mixed infections in poultry with other pathogenic bacteria, culminating in great economic losses in the poultry industry (2). More importantly, the main pathogenic APEC is highly homologous to the *E. coli* strain that causes human neonatal meningitis, so it poses a potentially serious risk to public health (3).

Iron is an important elemental metal involved in the metabolism of living organisms, including bacteria, and the maintenance of iron homeostasis in bacterial cells is extremely important (4). The concentration of iron that can be absorbed in the mammalian body is very limited. The concentration in the blood is the highest, but it is only 10^−25^ mol/L, which is far lower than the 10^−6^ mol/L concentration required for bacterial survival. After the bacteria infect the host, they inevitably need to compete with the host for iron and evolve to form a perfect iron uptake system in the long-term adaptation process mainly including the heme uptake system and siderophores. The heme iron uptake system mainly produce heme by bacteria to uptake iron from the host's hemoglobin. However, the iron uptake system of APEC produces relatively little heme and mainly produces siderophores to uptake iron. The siderophores mainly include Yersiniabactin, Enterobactin, Salmochelin, and Aerobactin. The iron-binding complexes mainly include: Yersiniabactin, Enterobactin, Salmochelin, and Aerobactin. These siderophores can combine with the free iron in the host to form an iron chelate, which is then recognized by the relevant receptors on the surface of the bacteria and enters the bacteria to achieves biological functions through the ABC transport system, TonB transport system, etc. Simultaneously, the siderophores can be re-released into the host environment for recycling. However, Excessive iron enriching in the bacteria will produce active oxygen and superoxide ions through the Fenton reaction and damage the bacteria indiscriminately. Therefore, the corresponding negative feedback regulation also exists in the bacteria, which mainly uses the iron absorption regulator protein Fur to exercise the regulatory function of iron homeostasis to prevent the bacteria from “iron poisoning.”

In 1981, Hantke first discovered the Fur protein in *E. coli*. In 1999, Escolar elucidated the role of Fur protein in maintaining iron homeostasis. The primary function of Fur is to maintain the iron homeostasis of bacteria, including the absorption, utilization, storage transportation and reduction of iron in bacteria (5). Fur is a homodimeric protein with a size of 17 KD and contains special metal binding sites. The Fur protein binds to an AT-rich sequence in the promoters of its target genes to regulate their expression. This sequence is often referred to as the “Fur box.” The classic Fur box sequence in *E. coli* is 5′-GATAATGATAATCATTATC-3′ (6), but experiments have shown that the site is not conserved. The target site sequence differs across different bacteria, but basically maintains 50–80% similarity to the classic sequence (7). In *E. coli*, the expression of 35 genes related to iron uptake is related to Fur. Changes in the concentration of iron ions in the environment directly affect the regulation of Fur, thereby affecting the synthesis of siderophores and the expression of outer membrane receptors. Fur also can regulate genes unrelated to bacterial iron uptake such as *flbB, fumC, metA, nohB, sodA*, etc., involving respiration, TCA cycle, DNA synthesis, flagella-mediated chemotaxis, etc. For instance, in Salmonella typhimurium, the Fur mutant has a significantly lower survival rate compared with the wild-type strain in an acidic environment.

The absorption and utilization of iron by bacteria is mainly regulated by the iron absorption regulatory protein Fur, but how Fur is regulated until the discovery of sRNA-RyhB was reasonably explained. In 2002, a non-coding RNA, RyhB, was identified and shown to be negatively regulated by Fur (8). In *E. coli*, RyhB is located between RyhX and RyhY with a length of 322 bp. Its structure consists of three parts including the binding domain that is reversing complementary to the target mRNA, the binding domain of the chaperone protein Hfq, and a transcription termination end that does not depend on ρ factor. The function of RyhB is mainly reflected in the balance of iron metabolism in bacteria and the regulation of genes related to pathogenicity. When iron is rich, Fur is activated and RyhB is inhibited, bacterial genes encoding ferritin (such as *bfr*) and non-essential iron sulfur protein genes (such as the sdhCDAB operon, *sodB* gene, etc.) are expressed, and iron ion is consumed to maintain the Fe^2+^ ion concentration in the cell at the normal level. On the contrary, when in an iron-deficient environment, Fur protein is in an inactive state, the expression of genes involved in iron absorption, and transport such as ferritin increases and the iron uptake of bacteria increases (8). Although RyhB itself is regulated by *the* Fur protein, RyhB also participates in the regulation of Fur protein synthesis by binding to the uof of the sequence upstream from the *fur* mRNA, forming a delicate and complex regulatory loop (9). By detecting iron availability in the environment, Fur and RyhB directly or indirectly regulate many virulence determinants of pathogenic bacteria through other transcriptional regulators or by regulating the iron concentration in the bacterium. These virulence determinants include the invasion of host cells (10), toxin production (11), quorum sensing (12), environmental tolerance (13), biofilm formation, and mobility (14).

In this study, we used *fur-, ryhB*-, and *fur*/*ryhB*-deficient mutants, which were constructed with the lambda red recombinase system (15), to identify genes differentially expressed in them with transcriptomic analyses, and performed a bioinformatic analysis. We then used electrophoretic mobility assays (EMSAs) to study the effects of Fur and RyhB on certain toxic genes of APEC, providing a reference for future research into the pathogenicity of APEC.

## Materials and Methods

### Bacterial Strains and Growth Conditions

*E. coli* was normally cultured in Luria–Bertani (LB) medium (tryptone 10 g/L, NaCl 10 g/L and yeast extract 5 g/L) at 37°C, or on LB solid medium containing 1.2% agar, with or without continuous oscillation at 150 rpm under aerobic conditions. The deletion strains (AE17Δ*fur*, AE17Δ*ryhB*, and AE17Δ*fur*/*ryhB*) were constructed previously in our laboratory with the lambda red recombinase system (16). Simply talk about the knockout steps, The pKD3 plasmid, pKD46 plasmid, and pCP20 plasmid (presented by the Poultry Research Laboratory, Shanghai Veterinary Research Institute, Chinese Academy of Agricultural Sciences) were all extracted using TIANprep Mini Plasmid Kit (TIANGEN BIOTECH, Beijing, China). The pKD46 plasmid was electrotransformed into AE17 competent cells and used 20 mM L-arabinose (Biosharp, Hefei, China) to induce the expression of Exo, Bet, and Gam. The targeting fragment of fur homology arm containing chloramphenicol resistance using pKD3 plasmid as template and P1, P2 primers (Table 1) to amplify was electrotransformed into AE17 competent cells containing pKD46 plasmid. The temperature-sensitive plasmid pCP20 containing Flp recombinase was used to eliminate the chloramphenicol resistance gene fragment. The construction of AE17Δ*ryhB*, and AE17Δ*fur*/*ryhB* is similar to AE17Δ*fur*.

**Table 1:** Primers and sequences used in this study.

**Name**	**Primer**	**Sequence (5′-3′)**	**Usage**
P1	pKD3-*fur*-F	TTATTTGCCTTCGTGCGCGTGCTCATCTTCGCGGCAATCGGTGTAGGCTGGAGCTGCTT	*fur* knockout
P2	pKD3-*fur*-R	ATGACTGATAACAATACCGCCCTAAAGAAAGCTGGCCTGACATATGAATATCCTCCTTAGTTC	
P3	pKD3-*ryhB*-F	AAAAAAAAAGCCAGCACCCGGCTGGCTAAGTAATACTGGAGTGTAGGCTGGAGCTGCTT	*ryhB* knockout
P4	pKD3-*ryhB*-R	ATTGACTTTCAAATGCGAGTCAAATGCATTTTTTTGCAAACATATGAATATCCTCCTTAGTTC	
AE17-Fur	*fur*-XhoI-F	CCG*CTCGAG*TTATTTGCCTTCGTGCGCG	Fur protein expression
	*fur*-BamHI-R	CG*GGATCC*ATGACTGATAACAATACCGCCC	
p-primers	FliA	F: AGTACGGCTATTGAGTATA	EMSA
		R: TATACTCAATAGCCGTACT	
	FlhD	F: GGGGAAGATAAACATTCCT	
		R: AGGAATGTTTATCTTCCCC	
	RyhB	F: GATAATGATAATCATTATC	
		R: GATAATGATTATCATTATC	

### Bacterial Growth Curves

The growth of strains AE17, AE17Δ*fur*, AE17Δ*ryhB*, and AE17Δ*fur*/*ryhB* was assayed in LB broth. Briefly, cultures were prepared in 20 ml of LB broth and cultured at 37°C with shaking at 180 rpm for 12–16 h. The optical density at a wavelength of 600 nm (OD_600_) of each strain was measured with spectrophotometry. The cultures were diluted with 100 ml of LB broth to achieve an approximate initial concentration of OD_600_ = 0.03 at the starting time (0 h). The bacterial cultures were incubated at 37°C with shaking at 180 rpm, and OD_600_ was monitored every hour for 22 h.

### Bacterial Motility Test

Bacterial motility was detected as previously described (26.). Briefly, LB medium was inoculated with the wild-type (WT), AE17Δ*fur*, AE17Δ*ryhB*, or AE17Δ*fur*/*ryhB* strain and incubated at 37°C for 12–16 h. The cultures were then transferred to 300 ml of LB liquid medium in a ratio of 1:100, grown at 37°C without shaking until OD_600_ = 1.0, and then centrifuged at 1,000 × g for 10 min. The medium was discarded and the bacteria were washed gently three times with phosphate-buffered saline (PBS). OD_600_ was adjusted to 2.0 with sterile PBS to concentrate the bacteria in the liquid. An aliquot (2 μL) of the bacterial suspension was pipetted onto the center of the motility test medium, and placed in a 37°C incubator for 6–8 h to observe the bacterial movement. The diameter of bacteria swimming ring was measured with a scale and photographed by Nikon camera (Nikon, Japan). To further verify bacterial motility, we statically cultured WT, AE17Δ*fur*, AE17Δ*ryhB*, or AE17Δ*fur/ryhB* in LB medium at 37°C for 12–16 h, pelleted at 1,000 × g for 5 min, resuspended in PBS, placed on copper mesh, and then we observed their flagella using a 200 kV Field Emission Transmission Electron Microscope (FEI Company, Hillsboro, USA).

### RNA Sequencing, Library Generation, and Bioinformatic Analysis

RNA sequencing, library construction, and a bioinformatic analysis were performed by Novogene, Beijing, China. After the RNA was quality tested, 3 μg of RNA per sample was used as the input material for the RNA sample preparation. Sequencing libraries were generated using the NEBNext® Ultra™ Directional RNA Library Prep Kit for Illumina® (New England Biolabs, Ipswich, MA, USA), according to the manufacturer's instructions, and index codes were added to attribute the sequences to each sample. The total RNA was examined with a NanoDrop 2000 spectrophotometer (Thermo Fisher Scientific, Waltham, MA, USA) and an Agilent Bioanalyzer 2100 (Agilent Technologies, Santa Clara, CA, USA). Sequencing libraries were generated according to the manufacturer's recommendations and the library fragments were purified with the AMPure XP system (Beckman Coulter, USA). Library quality was assessed with the Agilent Bioanalyzer 2100 system.

The clustering of the index-coded samples was performed with the cBot Cluster Generation System using TruSeq PE Cluster Kit v3-cBot-HS (Illumina), according to the manufacturer's instructions. After cluster generation, the libraries were sequenced on the Illumina HiSeq platform (Illumina, San Diego, CA, USA) and paired-end reads were generated. Finally, Gene Ontology (GO) and Kyoto Encyclopedia of Genes and Genomes (KEGG) enrichment analyses of the differentially expressed genes were performed. The raw RNA-seq data have been deposited in the National Center for Biotechnology Information gene expression database (https://www.ncbi.nlm.nih.gov) under SRA accession numbers: PRJNA682026

### Purification of Fur Protein

The *fur* open reading frame (ORF) was amplified from the WT genomic DNA with PCR using primers AE17-Fur (Table 1), cloned into the expression vector pET28a(+) (the protein expression vector pET-28a is stored in our laboratory), and then used to transform chemically competent *E. coli* DH5α cells. The cells were then plated on LB agar containing 50 μg/mL kanamycin. The positive colonies were selected and the recombinant plasmid pET–Fur was extracted and used to transform *E. coli* BL21(DE3) cells. The transformants were grown in 200 ml of LB medium at 37°C to an OD_600_ of about 0.3, and then transferred to 16°C. Protein expression was induced overnight with a final concentration of 0.6 μg/ml isopropyl-β-d-1-thiogalactopyranoside (IPTG). The cells were collected by centrifugation and washed twice with PBS buffer (137 mM NaCl, 2.7 mM KCl, 10 mM Na_2_HPO_4_, 2 mM KH_2_PO_4_, and pH 7.4). The cells were resuspended in 20 ml of lysis buffer (20 mM Tris, 1 M NaCl, and pH 8.0), sonicated for 15 min, and centrifuged at 9,600 × g for 10 min at 4°C. The supernatant was applied to a column filled with 2 ml of Ni-NTA agarose solution (Trans-gen Biotech, Beijing, Chinese), and allowed to combine at 4°C for 1 h. The column was then eluted with 10 mM, 50 mM, 100 mM, 200 mM, or 250 mM imidazole. Fur protein was finally eluted with 500 mM imidazole. The protein solution was stored in 10% glycerol at −80°C before use.

### Electrophoretic Mobility Shift Assays

The DNA fragments containing the gene promoters were amplified from the WT genomic DNA with PCR using p-primers (Table 1). The PCR products were purified and labeled with a digoxigenin (DIG) gel shift kit (Sigma-Aldrich, Shanghai, China), according to the manufacturer's instructions. The labeled DNA fragments were incubated with various amounts of purified McbR protein in 4 μl of 5 × binding buffer (100 mM Tris, 5 M NaCl, pH 8.0) at 25°C for 30 min. When required, unlabeled DNA fragments were added as competitive probes. After incubation, 5 μl of 5 × loading buffer containing bromophenol blue was added to the mixtures, which were then electrophoresed in a 4% native polyacrylamide gel in 0.5 × Tris-borate EDTA buffer (45 mM Tris-borate, 1 mM EDTA, pH 8.3). The band shifts were detected and analyzed according to the manufacturer's instructions.

### Statistical Analysis

All data were analyzed with one-way ANOVA with the statistical software SPSS (ver. 19.0, IBM Corp., Armonk, NY). The results are shown as means ± standard deviations (SD). A paired *t-*test was used for the statistical comparison of groups. The level of statistical significance was set at a *P*-value of ≤ 0.05.

## Results

### Deletion of *fur* and *RyhB* Did Not Affect Growth of AE17

**Figure 1:**
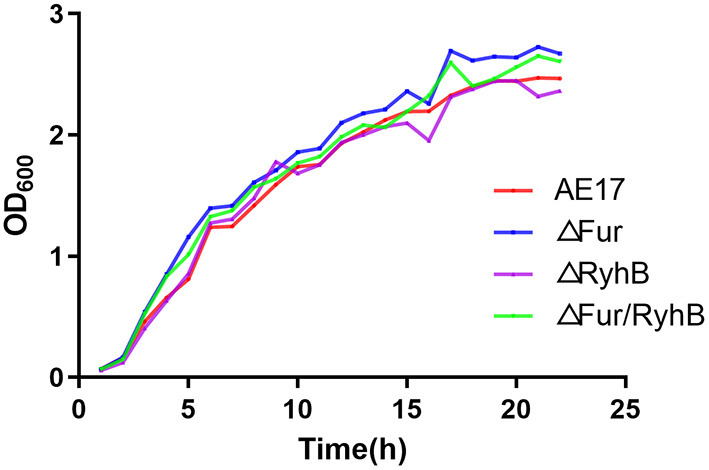
Growth curves of wild-type strain (WT), and mutant strains AE17Δ*fur*, AE17Δ*ryhB*, and AE17Δ*fur/ryhB* grown in LB broth at 37°C for 22 h with shaking. Growth was determined by measuring the optical cell density (OD) at 600 nm.

The growth curves of strains WT, AE17Δ*fur*, AE17Δ*ryhB*, and AE17Δ*fur*/*ryhB* showed that the deletion of *fur* and/or *ryhB* did not affect the growth of the avian pathogen, especially during its exponential growth phase. There was basically no difference in growth between the strains (Figure 1).

### Deletion of *fur* Reduces Bacterial Mobility

**Figure 2:**
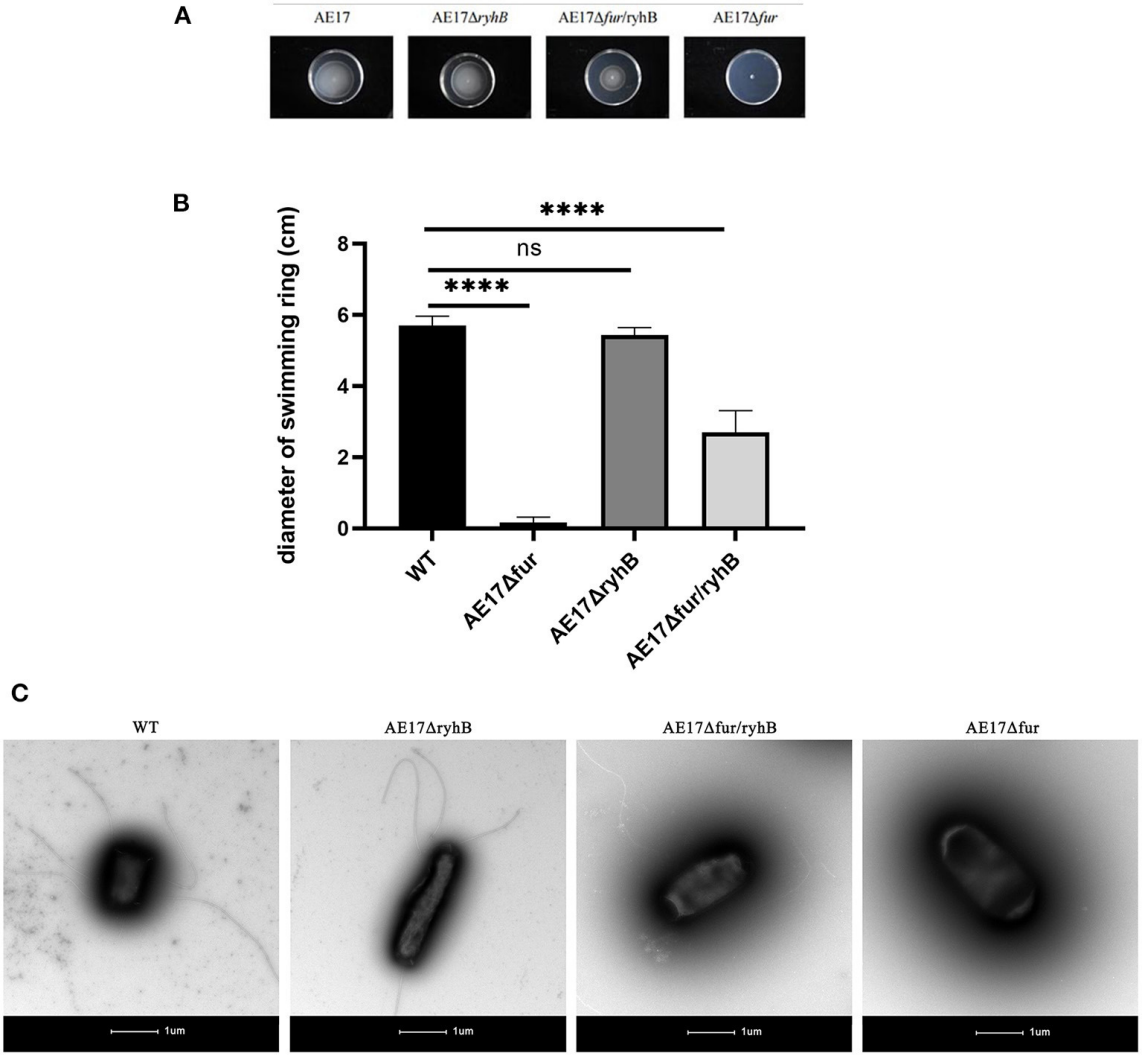
**(A)** Bacteria spread and formed circles in a sports medium containing 0.25% agar powder. **(B)** The diameter of the bacteria swimming ring (****P < 0.0001, ns = no significant). **(C)** Transmission electron microscopy images of WT, AE17Δ*ryhB*, AE17Δ*fur*, and AE17Δ*fur*/*ryhB*. Scale bars = 1 μm.

We compared the swimming ability of the mutants through motility experiments. The results showed that the mobility of AE17Δ*fur* was significantly lower than that of WT, and it had almost lost its ability to swiming. AE17Δ*ryhB* and WT did not differ significantly in their mobility. The mobility of AE17Δ*fur*/*ryhB* was also significantly weaker than that of WT, but was stronger than that of AE17Δ*fur* (Figures 2A,B). The flagella of WT, AE17Δ*ryhB*, AE17Δ*fur* and AE17Δ*fur*/*ryhB* further confirmed the above results. The results (Figure 2C) showed that AE17Δ*fur* had almost no flagella, and the number and length of flagella of AE17Δ*fur*/*ryhB* were greatly reduced compared to WT, and the number and length of flagella of AE17Δ*ryhB* were almost no reduced compared to WT.

### Transcriptional Profiles of Mutant Strains

**Figure 3:**
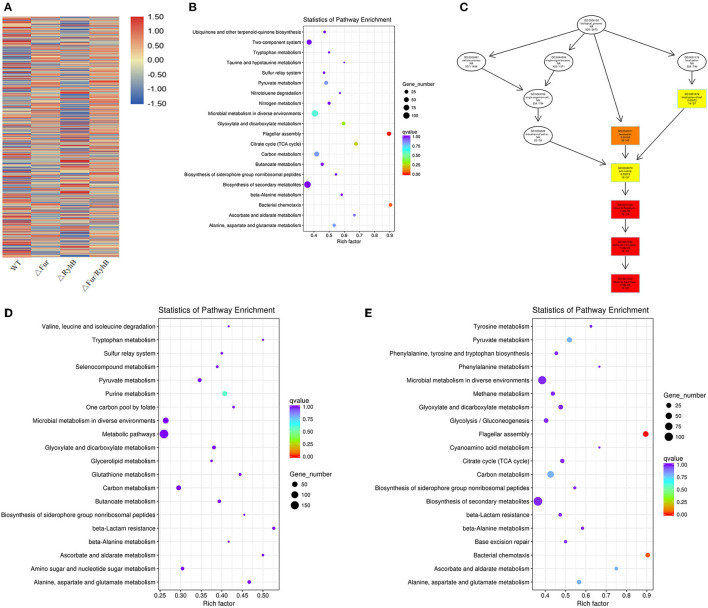
**(A)** Clusters of genes with the same or similar expression patterns, and the classification criterion is the relative expression level value log2 (ratios) of the DEGs for clustering. The 20 items that showed the largest changes in expression in **(B)** AE17Δ*fur*, **(C)** AE17Δ*ryhB*, and **(D)** AE17Δ*fur/ryhB* relative to WT in the KEGG analysis. The ordinate indicates the name of the pathway, the abscissa indicates the Rich factor, the size of the point indicates the number of DEGs in the pathway, and the color of the point corresponds to the *q-*value range. Directed acyclic graph (DAG) display of the most significantly DEGs. **(E)** Each node in the figure represents a GO term. The box contains the 10 most-enriched GO categories. The shade of the color represents the degree of enrichment: the darker the color, the greater the enrichment.

From the gene expression heat map, it can be seen that Fur negatively regulates RyhB in APEC (Figure 3A) and AE17Δ*fur* and AE17Δ*ryhB* had opposite regulatory effects on many genes (the same vertical axis has the opposite color). Differentially expressed genes (DEGs) were screened under the criteria |log2(fold change)| > 1 and *q* < 0.005. The results showed that the mutant AE17Δ*fur* had 1,286 DEGs, 649 of which were upregulated and 637 downregulated; AE17Δ*ryhB* had 1,001 DEGs, 459 of which were upregulated and 542 downregulated; and AE17Δ*fur/ryhB* had 1,266 DEGs, 668 of which were upregulated and 598 downregulated. GO and KEGG analyses of the DEGs showed that some of the DEGs affected by Fur were involved in oxidation–reduction activity and cofactor binding, but the most significant DEGs were enriched in pathways related to cell movement, such as pilus- and flagellum-dependent cell motility (Figures 3B,C). The DEGs affected by RyhB were mainly concentrated in the categories organic substance biosynthetic process, cellular biosynthetic process, and biosynthetic process (Figure 3D). The DEGs affected by Fur and RyhB combined were mainly concentrated in the oxidation–reduction process, but the genes related to cell motility were most significantly differentially expressed in the double mutant (Figure 3E). Reverse transcription (RT)–quantitative PCR (qPCR) was used to verify that the deletion of *fur* weakened the motor capacity of the bacterial strains by downregulating the transcription of flagellum-related genes, such as *fliC* (16).

### Promoter Prediction for Key Flagellar Genes *flhD* and *fliA*, and EMSA

**Figure 4:**
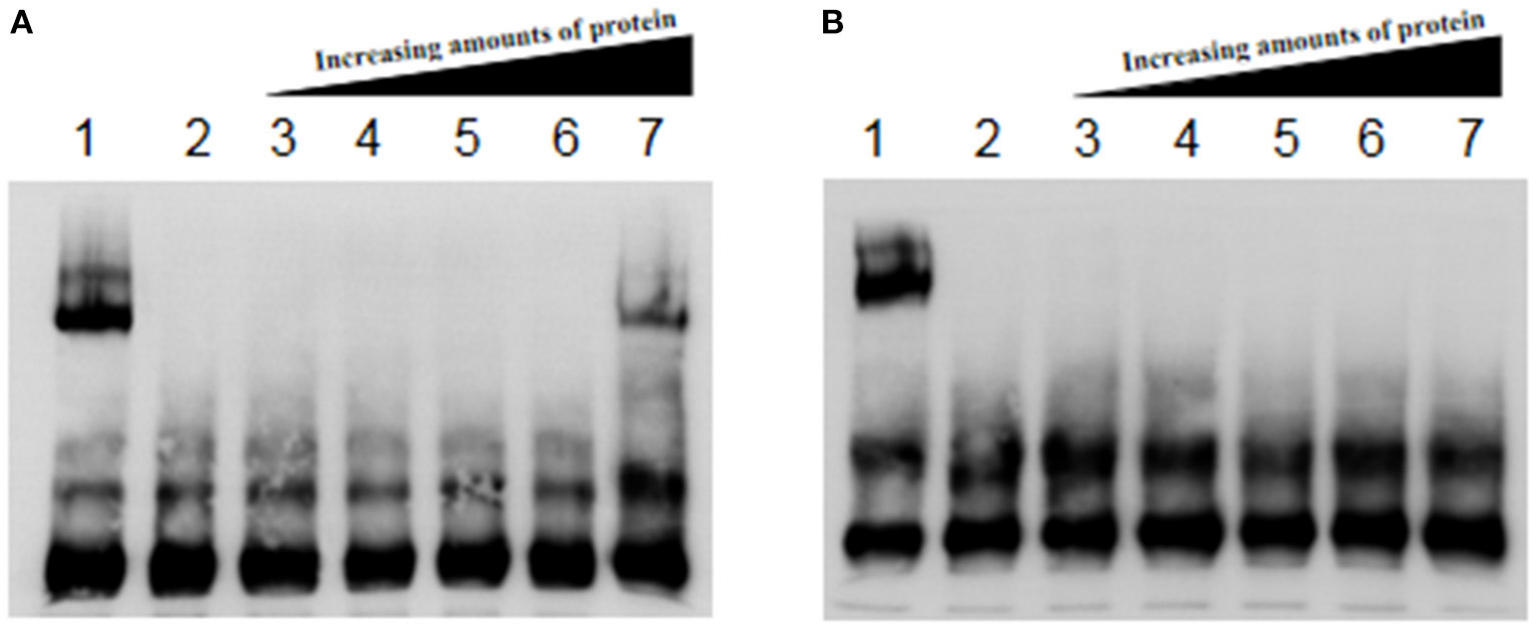
**(A)** Fur protein binding reaction with *flhD* promoter. **(B)** Fur protein binding reaction with the *fliA* promoter. Lane 1 contains the Fur protein binding reaction with RyhB as the positive control; lane 2 shows cold competition with unlabeled probe; and lanes 3–7 show the reactions of 0, 1, 2, 4, and 8 μM Fur protein with the labeled probe, respectively.

*flhD* and *fliA* are the key genes in the stepwise regulation of *E. coli* flagellum synthesis, so they were selected for further study. The complex of Fur protein and its metal cofactor binds to the Fur-box (GATAATGATAATCATTAT) or similar sequences in the target gene promoter and thus plays a regulatory role in their expression. We predicted the promoter regions of *flhD* and *fliA*, and screened for sequences similar to the Fur box. We hypothesized that Fur regulates flagellum synthesis by directly binding the promoter regions of *flhD* and *fliA* in AE17. Our results show that the Fur protein did not bind when its concentration was low, but when the protein concentration reached 8 μM, it bound to the *flhD* promoter sequence (Figure 4A). The Fur protein did not react with the promoter sequence of *fliA* (Figure 4B).

## Discussion

Previous studies have shown that the non-coding RNA RyhB is negatively regulated by Fur in *E. coli* K-12 (17). There have been many reports of related research in other bacteria, but the present study is the first to analyze the transcription profiles of *fur* and *ryhB* in APEC. Fur and RyhB are known to play important roles in the bacterial iron intake system, and also broadly affect the transcription of APEC genes. From the gene expression heat map, it can be seen that Fur also negatively regulates RyhB in APEC. In AE17Δ*fur* and AE17Δ*ryhB*, many genes displayed opposite regulatory effects (the same vertical axis had the opposite color). However, unexpectedly, the gene expression pattern in AE17Δ*fur*/*ryhB* was almost the same as that in AE17Δ*fur*, indicating that after the deletion of *fur*, the deletion of *ryhB* had no effect. The regulatory effect of RyhB on bacterial virulence is extensive, as reported in many studies (18). However, in this study, we found that it had no significant effect on a certain type of gene, which may require that the number of sequencing samples be increased to achieve.

In the RNA-Seq results, we found that *fur* and RyhB have significant regulatory effects on genes, especially flagellum-related genes. These differences were all regulated by *fur*. Thirty-four of the 36 genes in the flagellum-associated pathway of *E. coli* were downregulated in AE17Δ*fur*, and the same results were obtained in the motility tests. The deletion of *fur* even caused the bacteria to lose their ability to move. Motility is an important factor in bacterial invasion and adhesion to their host cells (19). It is also associated with bacterial chemotaxis and biofilm formation (20) and is therefore a very important virulence factor. Our experimental results also showed that the lack of *ryhB* compensated for the effect of *fur* loss on APEC motility, to a certain extent. A possible reason is that Fur and RyhB influence the synthesis of the APEC flagellum through the iron intake system. It has been reported that the synthesis of the flagellum requires iron (21). However, the excessive absorption and enrichment of iron ions produce reactive oxygen species (ROS), which cause serious cell damage. The absence of Fur destroys cellular iron homeostasis. In an environment rich in Fe^2+^ or Fe^2+^ inevitably causes cell damage and affects flagellum synthesis. When RyhB is removed, this problem is resolved because the iron steady state is restored.

The regulation of *E. coli* flagellum synthesis is divided into three levels. FlhD is a switch for flagellum synthesis, and FliA that in secondary regulation is the key protein controlling third level regulation (22). Fur probably has such a large effect on the flagellum because it directly regulates the expression of these two key genes. Our EMSA results confirmed that Fur binds the promoter of *flhD*, but not that of *fliA*, indicating that Fur directly regulates the expression of *flhD*, a gene of flagellum synthesis, thereby affecting the expression of other flagellum-related genes. Flagellin is also reportedly involved in the pathogenic process of the bacterium. Bacterial virulence requires that the flagellum-specific type III secretion system (F-T3SS) secretes effector proteins through different pathways and transmits them to the host cells to influence the relevant pathways of the host (23). FliC significantly enhanced the flagellin-specific immunoglobulin G (IgG) response after its overexpression in *Salmonella typhimurium*, and is a particularly attractive target for the development of candidate vaccines (24). The FlgE protein of *Pseudomonas aeruginosa* contains two sites at which it binds the host epithelial cell, suggesting that the flagellum plays an important role in the colonization of the host epithelial cells. Moreover, a recombinant FlgE protein stimulated the production of proinflammatory cytokines in human cell lines and rat lung organ cultures (24). The production of inflammatory cytokines suggests that flagellin-related proteins play a role in host–pathogen interactions. Based on the results of this study, the synthesis of the APEC flagellum can control Fur to reduce the pathogenicity of APEC. This strategy offers new options for the prevention and treatment of APEC-related diseases.

## Data Availability Statement

The datasets presented in this study can be found in online repositories. The names of the repository/repositories and accession number(s) can be found in the article/supplementary material.

## Author Contributions

LN wrote and revised the manuscript. WC performed the experiments. XC and ZL assisted in manuscript writing. JR and FL performed part of the experiments. KQ provided funds and resource assistance. JT designed the experiments and provided funds. All authors contributed to the article and approved the submitted version.

## Funding

This work was supported by grants from the National Natural Science Foundation of China (grant nos. 31972644 and 31502038) and the 2020 University Excellent Talents Support Program (gxyqZD2020009).

## Conflict of Interest

The authors declare that the research was conducted in the absence of any commercial or financial relationships that could be construed as a potential conflict of interest.

## Publisher's Note

All claims expressed in this article are solely those of the authors and do not necessarily represent those of their affiliated organizations, or those of the publisher, the editors and the reviewers. Any product that may be evaluated in this article, or claim that may be made by its manufacturer, is not guaranteed or endorsed by the publisher.
